# Sleep Quality in Medical Students of a Portuguese University: a cross-sectional Study

**DOI:** 10.1192/j.eurpsy.2023.606

**Published:** 2023-07-19

**Authors:** R. Gonzaga, J. Brás, A. P. Costa, C. Peixoto, J. Fialho

**Affiliations:** ^1^Faculty of Health Sciences, University of Beira Interior, Covilhã; ^2^Psychiatry and Mental Health, Centro Hospitalar Tondela Viseu; ^3^Escola Superior de Tecnologia e Gestão de Viseu, Viseu, Portugal

## Abstract

**Introduction:**

Sleep is a complex physiological process present in all living beings, performing essential functions for various biological functions. The prevalence of sleep disorders has increased exponentially, as well as studies relating to sleep patterns of the general population.

University students are especially vulnerable to a decrease in sleep quality, particularly medical students. Even so, the literature on sleep quality in medical students is scarce, especially when referring to Portugal, where studies are almost non-existent.

**Objectives:**

To evaluate sleep quality in medical students and to analyze the differences in sleep quality according to age, sex, cohabitation and physical activity. It is also intended to compare the sleep quality of medical students throughout the various phases of the medical course.

**Methods:**

This is a cross-sectional study involving medical students at the University of Beira Interior, Covilhã, Portugal. All medical students were invited to complete the Pittsburgh Sleep Quality Index (PSQI), which has been validated for the portuguese population. First, the scores obtained in each of the components of the PSQI and the global PSQI score were analyzed for the global population. Lastly, the global PSQI score was correlated with each of the sociodemographic variables to verify the existence of a statistically significant relationship.

**Results:**

296 students completed the instrument. Of these, 62.2% classify their sleep quality as good; 42.4% scored 2 in the sleep latency component; 50% reported sleeping 6 to 7 hours; 73.9% stated an adequate sleep efficiency; 85.5% mentioned few or no sleep disturbances; 83,8% said they never used sleep medication; and 60.8% had low or no sleepiness or daytime dysfunction.

As for the overall PSQI score, 72.6% of the students had a score greater than 5, indicating a poor quality of sleep. 74.7% of female respondents have a low quality of sleep, as well as 67.7% of male respondents. Likewise, 91.3% of students who live alone have poor sleep quality, as well as 76.8% of those living with family members and 69.8% of those living with colleagues.

Regarding the course year, 82.4% of the first-year students report a poor quality of sleep, as well as 77.5% of the second-year students, 72.1% of the third-year students, 77.8% of the fourth-year, 65.8% of the fifth-year students and 71.4% of the sixth-year students.

**Conclusions:**

Medical students seem to be more likely to have poor sleep quality, especially when compared to other university students. Thus, further studies are needed to prove this susceptibility as well as therapeutic interventions aimed at improving sleep parameters.

**Disclosure of Interest:**

None Declared

